# Electro-Thermal Bath

**Published:** 1877-10

**Authors:** 


*Therapeutic use of Faradaic and Galvanic Currents in the Electro-Thermal Bath with History of Cases.* By JUSTIN HAYES, M. D. Chicago: Jansen, McClurg & Co., 1877. pp. 112. Price $1.25.

Our author is confident of the great therapeutical power of electricity, especially when used in the bath. In the preface he says that after almost daily use of the Electro-Thermal Bath for over fifteen years, he concludes: "*I am confident that as an auxiliary in the treatment of diseases of women, it is a boon of greater value than has been discovered during the last fifty years.*" The italics are his own. After a short description of the bath, the general currents in the Electro-Thermal bath, and the manner in which it is to be used, he devotes the remainder of the work to the report of cases, twenty in number, illustrating nearly every disease that can be benefited by its use. We can recommend this work to those who wish to make use of the bath in the treatment of disease, but for ourselves, cannot entirely agree with the author in the unfailing power of the Electro-Thermal Bath. C.

